# Role of p53 mutation in the effect of boron neutron capture therapy on oral squamous cell carcinoma

**DOI:** 10.1186/1748-717X-4-63

**Published:** 2009-12-11

**Authors:** Yusei Fujita, Itsuro Kato, Soichi Iwai, Koji Ono, Minoru Suzuki, Yoshinori Sakurai, Ken Ohnishi, Takeo Ohnishi, Yoshiaki Yura

**Affiliations:** 1 Department of Oral and Maxillofacial Surgery, Osaka University Graduate School of Dentistry, Osaka, Japan; 2 Particle Radiation Oncology Research Center Laboratory, Research Reactor Institute, Kyoto University, Osaka, Japan; 3 Department of Biology, School of Medicine, Nara Medical University, Nara, Japan

## Abstract

**Background:**

Boron neutron capture therapy (BNCT) is a selective radiotherapy, being effective for the treatment of even advanced malignancies in head and neck regions as well as brain tumors and skin melanomas. To clarify the role of p53 gene, the effect of BNCT on oral squamous cell carcinoma (SCC) cells showing either wild- (SAS/neo) or mutant-type (SAS/mp53) p53 was examined.

**Methods:**

Cells were exposed to neutron beams in the presence of boronophenylalanine (BPA) at Kyoto University Research Reactor. Treated cells were monitored for modulations in colony formation, proliferation, cell cycle, and expression of cell cycle-associated proteins.

**Results:**

When SAS/neo and SAS/mp53 cells were subjected to BNCT, more suppressive effects on colony formation and cell viability were observed in SAS/neo compared with SAS/mp53 cells. Cell cycle arrest at the G1 checkpoint was observed in SAS/neo, but not in SAS/mp53. Apoptotic cells increased from 6 h after BNCT in SAS/neo and 48 h in SAS/mp53 cells. The expression of p21 was induced in SAS/neo only, but G2 arrest-associated proteins including Wee1, cdc2, and cyclin B1 were altered in both cell lines.

**Conclusion:**

These results indicate that oral SCC cells with mutant-type are more resistant to BNCT than those with wild-type p53, and that the lack of G1 arrest and related apoptosis may contribute to the resistance. At a physical dose affecting the cell cycle, BNCT inhibits oral SCC cells in p53-dependent and -independent manners.

## Background

Oral squamous cell carcinoma (SCC) patients are generally treated with surgery in combination with radiation therapy and/or chemotherapy [1,2].

Ionizing radiation (IR) directly damages DNA by causing single- and double-stranded breaks. p53 is a central mediator of the response to DNA damage and cell stress, therefore, it is expected to play a role in determining the sensitivity of tumors to apoptotic stimuli such as radiation or cytotoxic drugs [3-6].

Boron neutron capture therapy (BNCT) is a binary modality: Boron-10 (^10^B)-enriched compounds such as boronophenylalanine (BPA) and borocaptate sodium are administered at first, followed by irradiation with thermal neutrons. ^10^B to captures thermal neutrons leads to the nuclear reaction ^10^B (n, α) ^7^Li. Both released particles, an α (^4^He) particle and lithium (^7^Li) nucleus have high linear energy transfer (LET) properties and short path lengths in water of 5-10 μm. If the boronated compounds selectively accumulate in the tumor, BNCT can be used to selectively destroy tumor cells [7,8]. It has been shown that BNCT is effective for the treatment of advanced malignancies in head and neck regions as well as brain tumors and skin melanomas [9-12].

The level of localized DNA damage caused by IR is believed to increase with elevating LET values of radiation. Cell inactivation induced by IR with different LET's has been analyzed, and many studies have shown that high LET radiation including carbon-ion beams is more effective than low LET X-rays and gamma rays regarding the yield of apoptosis and reproductive death [13-16]. Carbon-ion beams have been reported to increase apoptosis in oral SCC and lung cancer cells regardless of the p53 status [17,18].

Approximately 50% of oral SCCs show a mutational change of p53 [19,20]. Before the novel high LET radiation therapy BNCT is used more frequently for oral SCC, its effect on the cell cycle and the cytotoxic effect on oral SCC cells irrespective of the p53 status should be clarified. In the present study, we examined the effects of BNCT on the proliferation, cell cycle, and cell cycle-related proteins of oral SCC cells showing wild- or mutant-type p53 with the same background and indicated the role of p53 in the suppressive effect of BNCT.

## Methods

### Cells

The oral SCC cell line SAS showed the phenotype of wild-type p53 on IR-induced signal transduction. SAS cells were transfected with the plasmid pC53-248 containing an mp53 gene (codon 248, from Arg to Trp) to produce a dominant negative mp53 protein, or with the control plasmid pCMV-Neo-Bam, which contains a neo-resistance marker. The stable transfectants SAS/mp53 and SAS/neo were used [21]. These oral SCC cell lines were cultured in Dulbecco's modified Eagle's medium supplemented with 10% fetal bovine serum, 2 mM L-glutamine, 100 μg/ml penicillin, and 100 mg/ml streptomycin at 37°C in a humidified atmosphere with 5% CO_2_.

### Boron compound and BNCT for cultured cells

^10^B-enriched (>98%) BPA was obtained from Boron Biologicals, Inc., (Raleigh, NC) and converted to a fructose complex following the method by Coderre et al. [22]. The concentration of the aqueous suspension of BPA was 250 mg/ml (21.28 mg ^10^B/ml).

For BNCT, cells were grown in flasks with a culture area of 25 cm^2 ^and treated with BPA at a ^10^B concentration of 50 ppm for 2 h. They were exposed to neutron beams in the presence of BPA at Kyoto University Research Reactor. Neutron fluence was measured by the radioactivation of gold foils on the front and back of the dishes, as described in previous studies [23,24]. The average fluence of thermal neutrons was 2.1 × 10^12 ^n/cm^2^, and the average flux was 2.3 × 10^9 ^n/cm^2^/s at 5 MW. Thermoluminescent dosimeters were used for gamma-ray dosimetry, and the total gamma ray dose was 0.00665 Gy. Thermal neutron fluence was converted to a dose, as described previously [24].

### Colony formation assay

Colony formation was performed as described previously [24]. Briefly, cells were dissociated with 0.05% trypsin and 0.02% EDTA, suspended in medium, and plated onto 60-mm dishes at a cell density yielding approximately 500 colonies per dish. The cells were cultured for 7 days, fixed in methanol, and stained with 1% crystal violet. Colonies composed of more than 30 cells were counted. The surviving cell fraction was determined by dividing the colony number of the treated culture by that of the non-irradiated control culture.

### 3-(4, 5-dimethylthiazol-2-yl)-2,5-diphenyltetrazolium bromide (MTT) assay

MTT assay was performed following the method by Mosmann [25]. Cells were seeded in 96-well plates at a density of 1 × 10^3 ^cells/well. At various intervals after BNCT, 10 μl of 5 mg/ml MTT solution was added to each well with 100 μl of medium, and cells were incubated at 37°C for 4 h. After the addition of 100 μl of 0.04 N HCl in isopropanol, the plates were mixed thoroughly to dissolve the dark blue crystals. The plates were read on a Benchmark Plus microplate spectrophotometer (Bio-Rad Laboratories, Hercules, CA) with a reference wavelength of 630 nm and a test wavelength of 570 nm. Background absorbance at 630 nm was subtracted from the 570 nm reading. The values for BNCT-treated cells were calculated as a ratio in relation to the untreated control cells. Data are presented as the means ± SD of six determinations.

### Flow cytometric analysis

Cells were dissociated and centrifuged, and the pellets were fixed in ice-cold 70% ethanol at -20°C overnight. Thereafter, the cells were washed twice with ice-cold PBS and treated with 1 mg/ml RNase at 37°C for 30 min. After staining of cellular DNA with 50 μg/ml propidium iodide in PBS, cells were analyzed with a fluorescence-activated cell sorter (FACSort; Becton Dickinson, Mountain View, CA). The percentage of cells at different phases of the cell cycle was determined by employing Mod Fit LT software (Verity Software House, Topsham, ME). Based on an analysis of DNA histograms, the percentages of cells in sub-G1, G0/G1, S, and G2/M phases were evaluated.

### Hoechst staining

Cells were dissociated and fixed in PBS containing 1% glutaraldehyde for 2 h. After washing in PBS, cells were stained with 200 μM Hoechst 33342, mounted on slides, and visualized using a Nikon Microphot-FXA fluorescence microscope. The number of positive cells was counted in 3 samples, and the mean ± SD was determined.

### Immunoblot analysis

Cells were lysed in a buffer containing 20 mM Tris-HCl (pH 7.4), 0.1% sodium dodecyl sulfate, 1% TritonX-100, 1% sodium deoxycholate, and protease inhibitor cocktail. After sonication, cells were centrifuged at 15,000 × g for 10 min at 4°C, and the supernatant was harvested. Protein (20 μg) was separated through polyacrylamide gel electrophoresis and transferred to a polyvinylidene fluoride membrane by electroblotting. The membrane was probed with antibodies, and antibody-binding was detected using an enhanced chemiluminescence kit (Amersham Life Science, Arlington Heights, IL) according to the manufacturer's instructions. The antibodies used were as follows: mouse monoclonal antibodies against p53, p53 phosphorylated at serine-15, p21, cyclin B1, and β-actin, and rabbit polyclonal antibodies against Wee 1 and cdc2 phosphorylated at tyrosine -15. Antibodies against p53 and β-actin were obtained from Oncogene (San Diego, CA) and Sigma (St.Louis, MO), respectively. Those for Wee1 and cyclin B1 were from Upstate (Lake Placid, MA). Other antibodies were from Cell Signaling Technology (Beverly, MA). The β-actin expression was assessed to ensure protein loading.

### Statistical analysis

The mean number of apoptotic cells was analyzed using the unpaired Student's *t-*test. A *P*- value < 0.05 was considered to be significant.

## Results

### Suppression of the colony formation of oral SCC cells by BNCT

**Figure 1:**
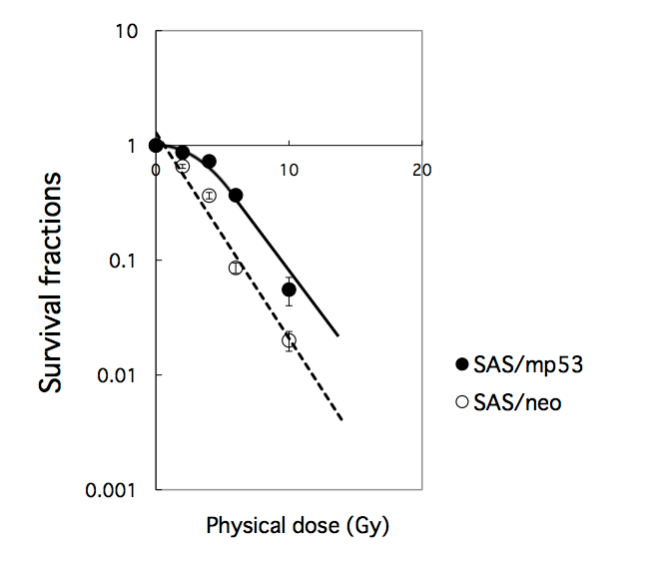
**Suppression of the colony formation of oral SCC cells by BNCT**. SAS/neo and SAS/mp53 cells were treated with BNCT, and survival fractions were assessed based on colony formation.

SAS/neo and SAS/mp53 cells were treated with BNCT, and the survival ratios were calculated based on colony formation. In both cell lines, the survival ratios decreased in a dose-dependent manner, but SAS/neo were suppressed more strongly than SAS/mp53 cells. At a dose of 6 Gy, the survival fractions of SAS/neo and SAS/mp53 cells were 8 and 36%, respectively (Figure 1).

### Suppression of the proliferation of oral SCC cells by BNCT

**Figure 2:**
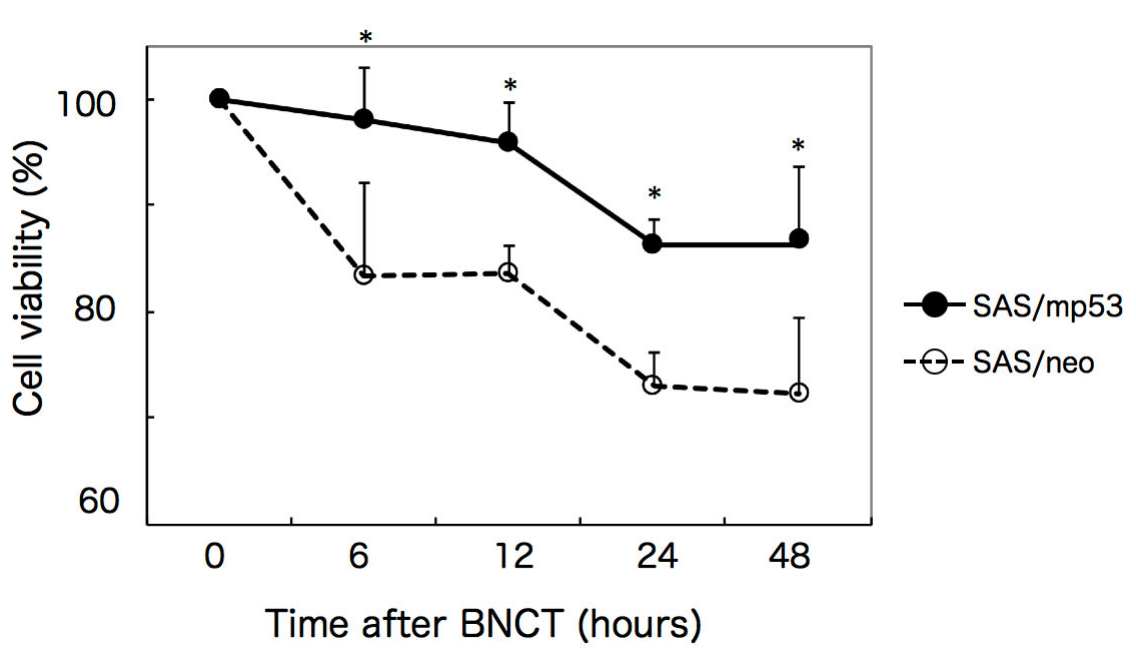
**Suppression of the proliferation of oral SCC cells by BNCT**. SAS/neo and SAS/mp53 cells were treated with BNCT, and cell viability was measured by the MTT assay. The cell viability of untreated cells was also measured and used as a control. *p < 0.01, SAS/neo vs. SAS/mp53.

To determine the effect of BNCT on the proliferation of cells, SAS/neo and SAS/mp53 cells were treated with BNCT at a dose of 6 Gy. After incubation for 6, 12, 24, and 48 h, cell viability was measured by employing the MTT assay. When the BNCT-treated cultures were compared with those of untreated controls, the percentage of viable cells was decreased in both cell lines. The rates of viable SAS/neo and SAS/mp53 at 48 h after BNCT were 72 and 86% of untreated controls, respectively (Figure 2), showing a significant difference (P < 0.01).

### Induction of cell cycle arrest by BNCT

**Figure 3:**
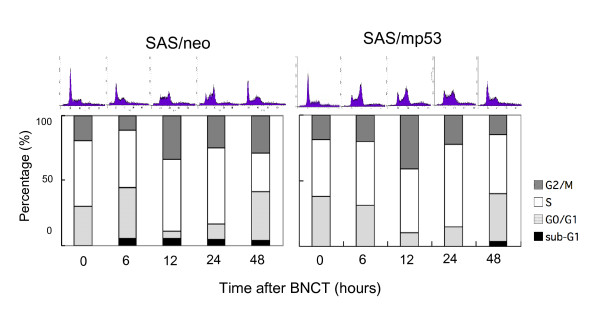
**Induction of cell cycle arrest by BNCT**. **A**. SAS/neo and SAS/mp53 cells were treated with BNCT and then subjected to flow cytometric analysis. **B**. Based on an analysis of DNA histograms, the percentages of cells in sub-G_1_, G_0_/G_1_, S, and G_2_/M phases were evaluated.

SAS/neo cells were treated with BNCT at a dose of 6 Gy and then subjected to flow cytometric analysis. Initially, the rate of SAS/neo cells in the G0/G1 phase was 30%, and it increased to 39% at 6 h after BNCT. At 12 h, it decreased to 6%, and cells in the G2/M phase were increased to 34%. Sub-G1 peaks, indicating apoptotic cells, appeared from 6 h after BNCT (Figure 3). In SAS/mp53 cells, however, there was no increase of G0/G1 phase cells at 6 h after BNCT; rather, they decreased slightly (Figure 3). At 12 h after BNCT, the proportion of cells in the G2/M phase was increased to 40%, indicating arrest at the G2/M checkpoint. A small sub-G1 population appeared at 48 h after BNCT.

### Measurement of apoptotic cells by nuclear staining

**Figure 4:**
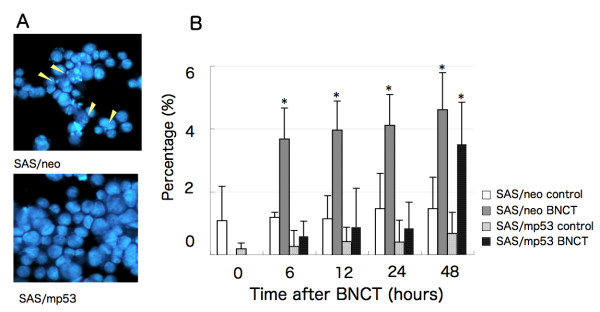
**Induction of apoptotic cells with the fragmentation of nuclear DNA by BNCT**. SAS/neo and SAS/mp53 cells were treated with BNCT, incubated for 48 h at 37°C, and stained by Hoechst 33342. The proportion of apoptotic cells was determined at various time points. *p < 0.01, SAS/neo vs. BNCT-treated SAS/neo; SAS/mp53 vs. BNCT-treated SAS/mp53.

Cell cycle analysis revealed the presence of a sub-G1 population, indicating apoptosis by BNCT. After treatment with BNCT, nuclear DNA was stained with Hoechst 33342, and cells showing nuclear fragmentation were determined (Figure 4A). In SAS/neo cells treated with BNCT, the proportion of apoptotic cells was elevated from 6 h as compared with untreated control cells, and reached 4.5% after incubation for 48 h (Figure 4B). The difference between SAS/neo and BNCT-treated SAS/neo was significant (p < 0.01). In the case of SAS/mp53, no apparent increase of apoptotic cells was observed early after BNCT, but the proportion increased to 3.5% at 48 h (Figure 4B). The difference between SAS/mp53 and BNCT-treated SAS/mp53 was significant (p < 0.01).

### The expression and/or phosphorylation of G1 checkpoint-related proteins by BNCT

**Figure 5:**
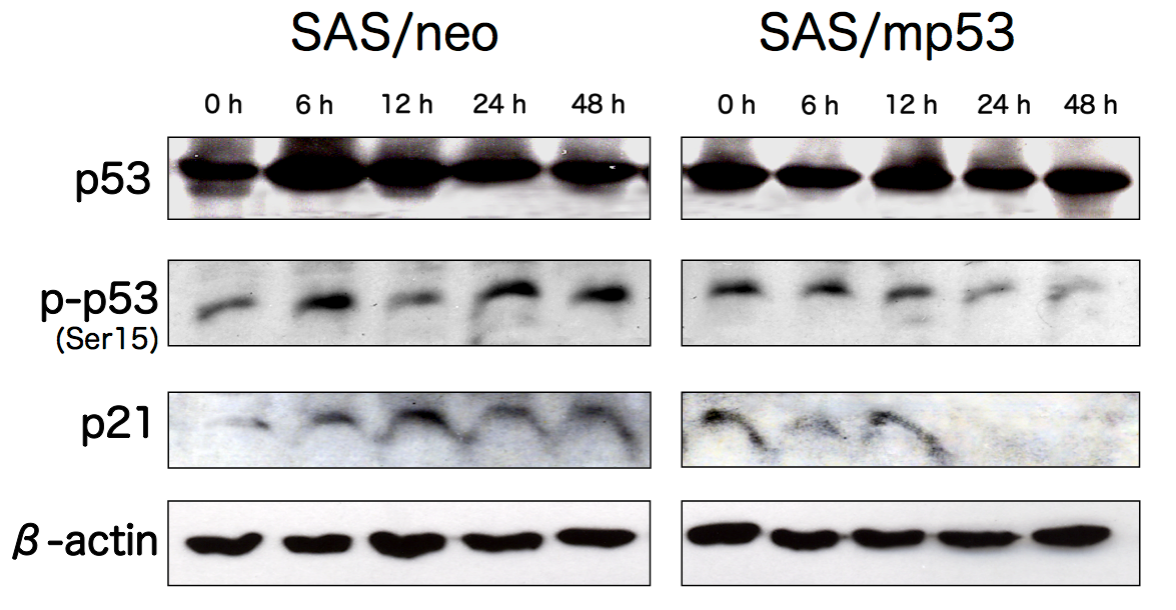
**Altered expression and/or phosphorylation of G1 checkpoint-related proteins by BNCT**. SAS/neo and SAS/mp53 cells were treated with BNCT, and the expression of p53 and p21 and phosphorylation of p53 were examined by immunoblot analysis.

In BNCT-treated SAS/neo cells, the expression of p53 increased and reached its maximum 6 h after BNCT. The elevation of phosphorylated p53 was observed at 6, 24, and 48 h after BNCT. An increased expression of p21 was observed from 6 h after BNCT (Figure 5). In SAS/mp53, the protein level of p53 was not specifically altered, but the phosphorylation decreased gradually after BNCT. The expression of p21 was also suppressed after BNCT in SAS/mp53 cells.

### The expression and/or phosphorylation of G2 checkpoint-related proteins by BNCT

**Figure 6:**
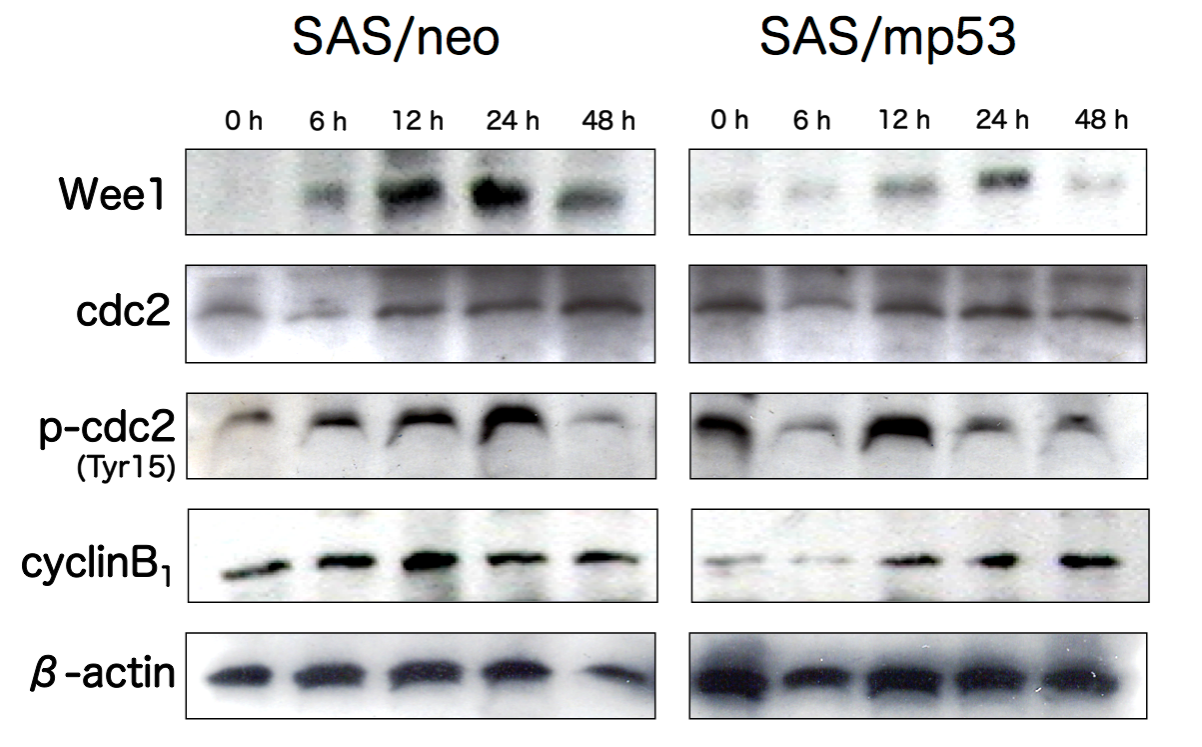
**Altered expression and/or phosphorylation of G2 checkpoint-related proteins by BNCT**. SAS/neo and SAS/mp53 cells were treated with BNCT, and the expression of Wee1, cdc2, and cyclin B1 and phosphorylation of cdc2 were examined by immunoblot analysis.

In SAS/neo cells, the expression of Wee1 was elevated from 12 to 24 h after BNCT, and rapidly decreased at 48 h (Figure 6). The protein level of cdc2 increased from 12 h after BNCT, and this was maintained until 48 h. An increase in the phosphorylation of cdc2 occurred at 12 h, indicating cell cycle arrest at the G2 checkpoint, and declined to the initial level at 48 h. Cyclin B1 that forms the cdc2/cyclin B1 complex was induced at 12 h after BNCT. In SAS/mp53 cells, the expression of Wee1 increased at 12 and 24 h after BNCT (Figure 6). Although the protein level of cdc2 was not specifically altered, cdc2 phosphorylation increased at 12 h after BNCT. The protein level of cyclin B1 increased from 12 h after BNCT, and this was maintained until 48 h.

## Discussion

It is considered that the presence of p53 mutation might reduce the effectiveness of radiotherapy, but studies comparing the presence or absence of p53 mutations in relation to the outcome following radiotherapy showed no consistent relationship [26-29]. Tumors with the wild-type p53 protein may lack a functional p53 response as a result of mutations affecting other genes that function in the same pathways as p53 [30]. It is difficult to clarify the role of p53 in each oral SCC cell line, and so we used known mutated oral SCC cell lines, SAS/neo and SAS/mp53, with the same background.

Studies on the correlation between the cytotoxic effect of BNCT and the p53 status are limited [31,32], but more studies are employing high LET carbon-ion beams. Indeed, Iwadate et al.[13] reported that high LET carbon-ion beams were more cytotoxic than low LET X-rays for glioma cells, and the effects of the carbon-ion beams were not dependent on the p53 gene status. Tsuboi et al. [15] reported that a glioblastoma cell line with p53 mutation was sensitive to carbon-ion beams as a wild-type p53 cell line at a high LET. In the present study, we performed colony formation assays, and confirmed that the effect of BNCT was more potent in SAS/neo than SAS/mp53 cells. We also examined the effect of BNCT using the MTT assay, and identified a difference between SAS/neo and SAS/mp53 cells regarding their proliferative potential after BNCT. The expression of functional p53 must be involved in BNCT-induced growth suppression and/or cell death.

p53 is a key factor that regulates the cell cycle checkpoint [4,6]. In this study, it was suggested that p53 plays an important role in G1 arrest in SAS/neo cells. Flow cytometric analysis revealed a transient accumulation in the G0/G population at 6 h after BNCT in SAS/neo cells. Thereafter, BNCT induced G2 arrest in both SAS/neo and SAS/mp53 cells. This indicates that BNCT induces cell cycle arrest at the G1 checkpoint only in SAS/neo cells. Tsuboi et al. [15] did not identify a marked increase of cells in the G1 phase in glioblastoma U87 MG cells with wild-type p53 as well as TK1 with mutant-type p53 after carbon-ion beam irradiation. BNCT may differ from carbon-ion beams in terms of its ability to induce cell cycle arrest at the G1 checkpoint.

When DNA damage by IR is irreparable, the activation of p53 leads to apoptosis via both transcription-dependent and -independent mechanisms. Aromando et al. [32] reported that BNCT-induced control of hamster cheek pouch tumors would be an inhibitory effect on DNA synthesis and apoptosis does not have a significant role in tumor control. Masunaga et al. [31] examined the effect of BNCT on SAS xenografts in nude mice. After BNCT, the tumor cells were dissociated and the cell suspension was cultured for colony formation, the detection of apoptotic cells, and a micronucleus assay. The peak of apoptosis was observed at 6 h after BNCT at low levels, irrespective of the p53 status, suggesting that apoptosis occurred early on. We also observed an increase in the sub-G1 population and nuclear fragmentation early after BNCT in SAS/neo cells, and the level was maintained thereafter. In SAS/mp53 cells, however, the increase in apoptosis occurred subsequent to G2 arrest. Thus, p53 seems to be responsible for G1 arrest-associated apoptosis. In the present study, p53 led to a significant but limited increase of apoptosis. Differently, in colony formation and MTT assays, p53 has a much stronger impact on the survival fraction and proliferation of treated cells. This indicates that apoptosis is a form of cell death induced by BNCT. So far, different types of cell death have been documented. They include apoptosis, autophagy, mitotic catastrophe, necrosis and senescence [33]. Especially, participation of mitotic catastrophe, necrosis and senescence in BNCT-treated cancer cells should be clarified.

p21 binds to and inhibits the cyclin-dependent protein kinases that drive the cell cycle, and is responsible for G1 arrest [34-36]. In SAS/neo cells, we found that the expression and phosphorylation of p53 was markedly enhanced from 6 h after BNCT, and this level was maintained for 48 h. We also detected a transient increase in the expression of p21 which inhibited the transition from the G1 to S phase. In SAS/mp53 cells, however, p21 was not induced, and neither G1 arrest nor the induction of apoptosis was observed. This indicates that p21 is associated with cell cycle arrest at G1 down-stream of the p53 pathway.

After BNCT, cells that escaped G1 arrest accumulated at G2 to prevent mitotic entry after potentially lethal DNA damage. Cdc2 protein kinase activity is required for the G2-to-mitosis transition in all eukaryotic cells. Cdc25 activates the cdc2/cyclin B1 complex by dephosphorylating inhibitory threonine-14 and thyrosine-15 residues of cdc2 [37-39]. This step is indispensable to mitosis after IR. Wee1 protein kinase allows cdc2 inactivation by phosphorylation of cdc2 on tyrosine -15 [40,41]. Matsumura et al. [42] reported that carbon-ion irradiation was associated with the overexpression of Wee1 and phosphorylation of cdc2, followed by the prolongation of G2 arrest and subsequent induction of apoptosis. Consistent with their results, we found that BNCT induced the expression of Wee1 and cyclin B1 and increased the phosphorylation of cdc2 in both SAS/neo and SAS/mp53 cells around 12 h after BNCT. Therefore, it can be stated that Wee1, cdc2, and cyclin B1 are associated with G2 arrest in a p53-independent manner.

Carbon-ion beams reportedly induce apoptosis in oral SCC and lung cancer cells regardless of the p53 status at a high LET [17,18]. Why high LET BNCT leads to the p53-dependent suppression of cell survival and induction of cell cycle arrest at the G1 checkpoint is unclear. Probably, each tumor cell would be equally exposed to carbon-ion beams. In the case of BNCT, however, the path lengths of high LET α and Li particles are very short, so that the LET would decrease markedly, even within a cell, being dependent on the distance from the cytoplasmic boron to the nuclear DNA [7,8]. This may generate a variety of intracellular LET values, and yield appropriate energy to induce cell cycle arrest at G1, if the cells have functional p53. It may also be ascribed to the characteristics of the cell lines used. Indeed, the survival curve of SAS/mp53 cells is not exponential, but a shoulder curve. The form of the curve suggests that the LET was not very high. If the mutation may influence the intracellular accumulation of BPA, it may heavily influence the LET of the radiation and relative biological effect.

In conclusion, oral SCC cells with mutant-type p53 were more resistant to the cell-killing effect of BNCT than those with wild-type p53 under the present experimental conditions. A functional p53 is required for the induction of apoptosis related to G1 arrest. BNCT inhibits oral SCC cells via p53-dependent and -independent mechanisms. Recent clinical studies have shown that the delivery of wild-type p53 to cancer cells with p53 mutations significantly increases their radiation sensitivity [43,44]. Adenoviral-mediated gene therapy is a reliable method to introduce the wild-type p53 gene [45,46]. Such an approach may be applicable to oral SCCs with mutated p53 to promote the efficiency of BNCT.

## Conflict of interests

The authors declare that they have no competing interests.

## Authors' contributions

YF carried out the experiments in the study and drafted the manuscript. IK provided the compound and carried out the experiments. SI carried out the experiments. KO participated in the design of reactor irradiation. MS helped the measurement of boron concentration. YS helped reactor irradiation. KO provided cell lines and participated in the design of the study. TO provided cell lines and participated in the design of the study. YY conceived of the study and participated in its design and coordination. All authors read and approved the final manuscript.
